# Fragments of urban forest in a large Amazonian city as habitats for potential Leishmania vectors

**DOI:** 10.1016/j.parepi.2026.e00504

**Published:** 2026-04-12

**Authors:** Yago Costa Vasconcelos dos Santos, Thiago de Matos Bezerra, Doralice Crisóstomo dos Reis, Rogério Valois Laurentino, Leonardo Miranda dos Santos, Ana de Nazaré Martins da Silva, Thiago Vasconcelos dos Santos, Edna Aoba Yassui Ishikawa

**Affiliations:** a Laboratory of Molecular and Cellular Biology, Center for Tropical Medicine, Federal University of Pará, Belém, Brazil; b Faculty of Geography, Institute of Philosophy and Human Sciences, Federal University of Pará, Belém, Brazil; c Laboratory of Virology, Institute of Biological Sciences, Federal University of Pará, Belém, Brazil; d Laboratory of Medical Entomology and Venomous Arthropods, Center for Tropical Medicine, Federal University of Pará, Belém, Brazil; e Parasitology Section, Evandro Chagas Institute, Ananindeua, Brazil

**Keywords:** Phlebotomine, *Leishmania*, Leishmaniasis, Amazon

## Abstract

**Objective:**

To characterise the phlebotomine sand fly fauna, including potential *Leishmania* vectors, in urban forest fragments within a leishmaniasis-endemic area of the Brazilian Amazon.

**Methods:**

The study was conducted in three sites: Parque Estadual do Utinga, Mata do Batalhão “Pedro Teixeira”, and Mata da Marinha. Ten CDC light traps were installed per site and operated from 18:00 to 06:00 between September 2014 and May 2016. Female sand flies were taxonomically identified and grouped into pools by species and collection site. Natural infection with *Leishmania* was assessed using polymerase chain reaction (PCR).

**Results:**

A total of 8465 phlebotomine sand flies were collected, with Mata do Batalhão “Pedro Teixeira” contributing the largest proportion. The assemblage was dominated by *Nyssomyia antunesi* (60.01%), followed by *Psychodopygus davisi* (18.01%) and *Bichromomyia flaviscutellata* (4.89%). No *Leishmania* DNA was detected.

**Conclusion:**

Despite the absence of detected infection, the high abundance and wide distribution of confirmed and putative vector species indicate that urban forest fragments in Belém provide favourable ecological conditions for *Leishmania* transmission. These findings underscore the importance of continued entomological surveillance and vector monitoring in urban environments.

## 1 Introduction

Phlebotomine sand flies (Diptera: Psychodidae) are arthropods of recognised medical importance, as they act as vectors of the genus *Leishmania* Ross, 1903 (Ryan et al., 1984), the aetiological agent of leishmaniasis (Araujo-Pereira et al., 2020). More than 1000 phlebotomine species are currently described worldwide, of which approximately 280 have been recorded in Brazil (Galati et al., 2025). In major Amazonian cities, urban forest fragments constitute ecosystems characterised by high biodiversity; however, progressive deforestation of primary forest areas may facilitate the displacement of vector species into peri-urban and urban settings, thereby enabling the establishment of urban transmission cycles of American Tegumentary Leishmaniasis (ATL) in the presence of infected reservoirs (Costa et al., 2024).

In the Brazilian Amazon, approximately 70% of the phlebotomine sand fly species recorded in Brazil have been reported, including several taxa potentially involved in the transmission of American tegumentary leishmaniasis (ATL), such as *Bichromomyia flaviscutellata* (Mangabeira, 1942), *Bichromomyia olmeca nociva* (Young & Arias, 1982), *Migonemyia migonei* (França, 1920), *Nyssomyia anduzei* (Rozeboom, 1942), *Nyssomyia antunesi* (Coutinho, 1939), *Nyssomyia umbratilis* (Ward & Fraiha, 1977), *Nyssomyia whitmani* (Antunes & Coutinho, 1939), *Psychodopygus complexus* (Mangabeira, 1941), *Psychodopygus davisi* (Root, 1934), *Psychodopygus wellcomei* Fraiha, Shaw & Lainson, 1971, and *Trichophoromyia ubiquitalis* (Mangabeira, 1942) (Dos Santos Teles Oliveira et al., 2024). Cases of ATL in the region are caused predominantly by *Leishmania* (*Leishmania*) *amazonensis* Lainson & Shaw, 1972, *Leishmania* (*Viannia*) *lainsoni* Silveira, Shaw, Braga & Ishikawa, 1987, *L.* (*V.*) *lindenbergi* Silveira, Ishikawa, de Souza & Lainson, 2002, *L.* (*V.*) *naiffi* Lainson & Shaw, 1989, and *L.* (*V.*) *shawi* Lainson, Braga, Souza, Póvoa & Ishikawa, 1989 (Gonçalves et al., 2020a). Forest fragments provide environmental conditions conducive to the maintenance of the *Leishmania* life cycle (Sánchez Uzcátegui et al., 2020; Pereira Júnior et al., 2019; Pinto et al., 2023; Carneiro et al., 2023). Such conditions may also be present in urban forested environments subject to sustained human circulation, including urban parks and woodland areas (Barroso et al., 2025), as well as in sites used for military training activities (Sánchez Uzcátegui et al., 2020; Lamattina et al., 2024).

High incidence rates of ATL result from multiple interacting factors, including the ecological success and expansion of phlebotomine populations supported by the abundance of vertebrate reservoirs and vectors, a hot and humid tropical climate, increased human exposure to sylvatic environments associated with deforestation and natural resource exploitation, unplanned urbanisation, and insufficiently structured preventive health policies (Portella and Kraenkel, 2021). These determinants are reflected in an estimated incidence of approximately 46.4 cases per 100,000 inhabitants (de Melo et al., 2024). Brazil maintains a universal public health system that implements, in a decentralised manner, the National Leishmaniasis Control Programme, which aims to reduce transmission and disease-related mortality through surveillance and monitoring of transmission areas. Such actions require systematic entomological surveillance, particularly in environments with active human circulation, including recreational and urban forested areas (Brasil. Ministério da Saúde. Secretaria de Vigilância em Saúde. Departamento de Vigilância das Doenças Transmissíveis, 2017). Within this framework, the present study aimed to investigate the occurrence and composition of phlebotomine sand flies in urban forest fragments located in the Belém Metropolitan Region (BMR), in the Brazilian Amazon, and to evaluate natural *Leishmania* infection through PCR-based molecular analysis.

## 2 Methods

### 2.1 Study design and setting

A cross-sectional, analytical, and descriptive study was conducted to investigate the phlebotomine sand fly fauna in three urban forest fragments in Belém, Pará State, Brazilian Amazon: Parque Estadual do Utinga, Mata da Marinha, and Mata do Batalhão “Pedro Teixeira” (the latter serving as a training area for the 2nd Jungle Infantry Battalion). The region has an equatorial climate, with mean annual temperatures of 26–32 °C, relative humidity around 87%, and average annual rainfall of ∼3000 mm, with a rainy season from January to May (Brasil. Instituto Brasileiro de Geografia e Estatística, 2022).

The study areas are designated conservation units comprising mainly terra firme rainforest, secondary (anthropogenically modified) forest, and seasonally flooded igapó forest, supporting high biodiversity. Vegetation includes medium- to large-sized trees, and fauna includes wild mammals such as medium- and large-sized rodents. Soils are rich in decomposing organic matter, creating suitable habitats for phlebotomine sand flies.

#### 2.1.1 Parque Estadual do Utinga - PEUT (1°24′39.55”S; 48°24′37.27”W)

A protected area of ∼1393 ha, mostly within Belém (99%) and partly in Ananindeua (1%). The park includes terra firme forest, secondary forest, and seasonally flooded igapó forest, with high faunal and floral diversity. Parque Estadual do Utinga is a public area for recreation, sports, and scientific research.

#### 2.1.2 Mata do Batalhão “Pedro Teixeira” - MBPT (1°24′45.15”S; 48°26′47.82”W)

A Brazilian Army training area with Amazonian vegetation partially impacted by military activities. The site hosts native fauna such as armadillos and marsupials and is located a few kilometers from Parque Estadual do Utinga. Surrounding urban neighborhoods with domestic animals create potential human–vector interfaces.

#### 2.1.3 Mata da Marinha - MM (1°23′29.70”S; 48°26′29.07”W)

A secondary forest remnant within a Navy-managed area, including upland and floodplain forest. It functions as an ecological corridor and refuge for urban wildlife, including birds, small mammals, and reptiles. The site faces anthropogenic pressures, including deforestation and urban development (e.g., widening of Rua da Marinha), which fragment habitats, affect ecosystems, and may increase human exposure to disease vectors.

Seventeen four-day sampling campaigns were conducted between September 2014 and May 2016: one in PEUT, two in MM and fourteen in MBPT. Phlebotomine sand flies were captured using ten CDC light traps per site, deployed in dense, closed-canopy forest at both ground level and in the canopy, with a minimum spacing of 10 m between traps. Traps operated nightly from 18:00 to 06:00.

### 2.2 Identification of phlebotomine sand flies

Specimens were morphologically identified according to Galati (Galati, 2018), and recorded by species, sex, date, and site of capture. Following identification, specimens were preserved in tubes containing absolute isopropanol (C₃H₈O). For DNA extraction and amplification by polymerase chain reaction (PCR), only females were grouped into pools by species and collection site in 1.5 mL tubes containing isopropanol, and screened for natural infection with *Leishmania*.

### 2.3 DNA extraction

Isopropanol was removed from the sand fly samples, which were subsequently washed in distilled water and transferred to 1.5 mL conical microtubes. Genomic DNA was extracted using the Wizard® Genomic DNA Purification Kit (Promega, A1125, Wisconsin, USA) following the manufacturer's protocol, with a modification consisting of extending the insect tissue lysis incubation time from 10 to 30 min. Extracted DNA was stored at −20 °C until further analysis.

### 2.4 Molecular quality assessment and *Leishmania* DNA detection

DNA extraction quality was evaluated by PCR amplification using primers CB3-PDR (5′-CA(T/C)ATTCAACC(A/T)GAATGATA-3′) and N1N-PDR (5′-GGTA(C/T)(A/T)TTGCCTCGA(T/A)TTCG(T/A)TATGA-3′), which amplify a 550 bp fragment of the mitochondrial cytochrome *b* (mtDNA) gene. Reaction mixtures were prepared according to Ready et al. (1997) (Ready et al., 1997) in a final volume of 15 μL containing 1× PCR buffer (Invitrogen, USA), 1.5 mM MgCl₂ (Invitrogen, USA), 60 μM of each deoxynucleotide triphosphate (dNTP), 100–500 ng of each primer (CB3-PDR and N1N-PDR), 1.5 U Taq DNA polymerase (Invitrogen, USA), and 50–250 ng of template DNA. PCR amplifications were performed in an MJ96+ Biocycler under the following conditions: initial denaturation at 94 °C for 3 min, followed by enzyme addition for hot start at 80 °C for 10 min; 30 cycles of denaturation at 94 °C for 30 s, annealing at 38 °C for 30 s, and extension at 72 °C for 1 min 30 s; and a final extension at 72 °C for 10 min.

Molecular detection of *Leishmania* DNA was performed by PCR using primers S1629 (5′-GGGAATTCAATAWAGTACAGAAACTG-3′) and S1630 (5′-GGGAAGCTTCTGTACTWTATTGGTA-3′), which amplify kinetoplast DNA (kDNA) minicircle sequences. These primers target a highly repetitive and conserved region, generating fragments of approximately 450 bp for *L.* (*L.) infantum chagasi* Cunha & Chagas, 1937, 350 bp for *L.* (*L.*) *amazonensis*, and 250 bp for species of the subgenus *L.* (*Viannia*) (Fernandes et al., 1994). PCR reactions followed a protocol adapted from Fernandes et al. (1994) (Fernandes et al., 1994). Each reaction was carried out in a final volume of 10 μL containing 10% dimethyl sulfoxide (DMSO), 1× PCR buffer (Invitrogen, USA), 1 mM MgCl₂ (Invitrogen, USA), 100 μM of each deoxynucleotide (dNTP), 100 pmol of each primer (S1629 and S1630), 2.5 U Taq DNA polymerase (Invitrogen, USA), and 50–250 ng of template DNA. Amplification was performed in a thermocycler under the following conditions: initial denaturation at 95 °C for 5 min; five cycles of denaturation at 95 °C for 30 s, annealing at 45 °C for 30 s, and extension at 65 °C for 1 min; followed by 35 cycles of denaturation at 95 °C for 1 min, annealing at 50 °C for 30 s, and extension at 72 °C for 1 min; with a final extension at 72 °C for 3 min. Positive controls consisted of genomic DNA extracted from reference strains of *L.* (*L*.) *infantum chagasi* (MCAN/BR/2005/M23485), *L.* (*Viannia*) *braziliensis* (MHOM/BR/1975/M2903), and *L.* (*L*.) *amazonensis* (IFLA/BR/1967/PH8).

### 2.5 Agarose gel electrophoresis

Amplified products were subjected to horizontal electrophoresis at 100 V and 50 mA for 1 h in Tris–acetate–EDTA (TAE) buffer (40 mM Tris-acetate, 1 mM EDTA; pH 8.0) using a 1% agarose gel stained with ethidium bromide (final concentration: 0.5 μg/mL). Samples were loaded with bromophenol blue tracking dye (0.25% bromophenol blue, 0.25% xylene cyanol, 30% glycerol). Gels were subsequently visualized and documented using an L-PIX Loccus photodocumentation system. Amplification products of the mini-exon gene were analyzed by electrophoresis in 1.5% agarose gels prepared in TAE buffer under the same electrophoretic conditions described above.

### 2.6 Data analysis

Sampling effort considered the total number of campaigns, the number of traps deployed, and the duration of trap operation in hours. The minimum natural infection rate (MIR) was estimated under the assumption that each positive pool contained a single infected sand fly. Following the approach proposed by Paiva et al. (2007) (Paiva et al., 2007), the MIR was calculated by dividing the number of pools testing positive for *Leishmania* DNA by the total number of sand flies analyzed and multiplying the result by 100. The infection rate was expressed as the number of infected individuals per 100 examined specimens.

### 2.7 Ethical considerations

This study did not require approval by a Research Ethics Committee, as it involved only the collection of insect specimens and did not include human participants or vertebrate animals. Access to the urban forest fragments was previously requested from the authorities responsible for site management, and all necessary permissions to conduct field activities were formally obtained.

## 3 Results

A total of 8465 phlebotomine sand flies were captured during the study period, with females predominating in all sampling areas. Overall, females represented 64.5% of the specimens (5463/8465), whereas males accounted for 35.5% (3002/8465), resulting in a female-to-male ratio of 1.8:1. The majority of specimens were collected in MBPT, where 7730 individuals were recorded, comprising 64.3% females and 35.7% males. In PEUT, 535 sand flies were captured, with females representing 61.8% of the total, while in MM, 200 specimens were collected, showing the highest female proportion (77.0%) among the study sites (Table 1).

**Table 1:** Distribution of female and male sandflies captured in the three urban forest fragments of this study.

	MBPT	PEUT	MM	Total
	n (%)	n (%)	n (%)	n (%)
Fêmea	4978 (64.3)	331 (61.8)	154 (77)	5463 (64.5)
Macho	2752 (35.7)	204 (38.2)	46 (23)	3002 (35.5)
Total n (%)	7730 (100.0)	535 (100.0)	200 (100.0)	8465 (100.0)

MBPT (Mata do Batalhão “Pedro Teixeira”); PEUT (Parque Estadual do Utinga); MM (Mata da Marinha).

The sampled fauna comprised 29 species distributed across 12 genera. The assemblage was strongly dominated by *Nyssomyia*, mainly due to the high abundance of *Ny. antunesi* (Coutinho, 1939), which alone represented 65.46% of all collected specimens. *Bichromomyia flaviscutellata* (Mangabeira, 1942) (5.33%) and *Ny. yuilli yuilli* (Young & Porter, 1972) (0.04%) occurred at considerably lower frequencies. The second most abundant genus was *Psychodopygus* (20.17%), largely represented by *Psychodopygus davisi* (19.65%), while the remaining species were recorded in low numbers. *Brumptomyia* accounted for 8.33% of the total captures but was not identified to species level. Other genera were comparatively less abundant, including *Evandromyia* (2.93%) and *Psathyromyia* (2.18%), whereas the remaining genera each represented less than 1% of the sampled fauna. Overall, the community structure was characterised by marked dominance of a few species alongside a large proportion of rare taxa (Table 2).

**Table 2:** Phlebotomine sand flies captured in three urban forest fragments of Belém metropolitan region, Pará state, Brazil.

Genus	Species	n (%)
*Brumptomyia*	–	705 (8.33)
*Nyssomyia*	*Nyssomyia antunesi*⁎	5080 (65.46)
	*Bichromomyia flaviscutellata*⁎	414 (5.33)
	*Nyssomyia yulli yulli*	3 (0.04)
*Psychodopygus*	*Psychodopygus davisi*⁎	1525 (19.65)
	*Psychodopygus geniculatus*	108 (1.40)
	*Psychodopygus ayrozai*⁎	65 (0.84)
	*Psychodopygus paraensis*⁎	5 (0.06)
	*Psychodopygus hirsutus hirsutus*⁎	3 (0.04)
	*Psychodopygus carrerai carrerai*	1 (0.01)
*Evandromyia*	*Evandromyia infraspinosa*	242 (3.12)
	*Evandromyia brachyphalla*	3 (0.04)
	*Evandromyia monstruosa*	2 (0.03)
	*Evandromyia pinottii*	1 (0.01)
*Psathyromyia*	*Psathyromyia barrettoi barrettoi*	111 (1.43)
	*Psathyromyia aragaoi*	1 (0.01)
	*Psathyromyia lutziana*	2 (0.03)
*Viannamyia*	*Viannamyia tuberculata*	37 (0.48)
	*Viannamyia furcata*	4 (0.05)
*Trichopygomyia*	*Trichopygomyia longispina*	33 (0.42)
	*Trichopygomyia dasypodogeton*	3 (0.04)
*Sciopemyia*	*Sciopemyia sordellii*	25 (0.32)
*Lutzomyia*	*Lutzomyia gomezi*⁎	11 (0.14)
	*Lutzomyia evangelistai*	6 (0.08)
	*Lutzomyia carvalhoi*	1 (0.01)
*Trichophoromyia*	*Trichophoromyia ubiquitalis*⁎	15 (0.19)
*Micropygomyia*	*Micropygomyia rorotaensis*	52 (0.67)
	*Micropygomyia trinidadensis*	4 (0.05)
	*Micropygomyia micropyga*	2 (0.03)
*Pintomyia*	*Pintomyia damascenoi*	1 (0.01)
**Total**		**8465 (100.00)**

⁎ Species of medical importance for *Leishmania* spp.

Among the subgenera identified, *Psychodopygus* exhibited the greatest species richness, comprising six species (*Lutzomyia davisi*, *Lutzomyia geniculata*, *Lutzomyia ayrozai*, *Lutzomyia paraensis*, *Lutzomyia hirsuta hirsuta*, and *Lutzomyia carrerai carrerai*). This was followed by *Evandromyia*, with four species (*Lutzomyia infraspinosa*, *Lutzomyia brachyphalla*, *Lutzomyia monstruosa*, and *Lutzomyia pinotti*); *Nyssomyia*, with three species (*Lutzomyia antunesi*, *Bichromomyia flaviscutellata*, and *Lutzomyia yulli yulli*); and the subgenus *Lutzomyia*, also with three species (*Lutzomyia gomezi*, *Lutzomyia evangelista*, and *Lutzomyia carvalhoi*).

Of the species collected, eight are recognised as being of medical importance: *Lutzomyia (Nyssomyia) antunesi*, *Lutzomyia (Psychodopygus) davisi*, *Lutzomyia (Nyssomyia) flaviscutellata*, *Lutzomyia (Psychodopygus) ayrozai*, *Lutzomyia (Trichophoromyia) ubiquitalis*, *Lutzomyia (Lutzomyia) gomezi*, *Lutzomyia (Psychodopygus) paraensis*, and *Lutzomyia (Psychodopygus) hirsuta hirsuta* (Table 3).

**Table 3:** *Lutzomyia* species captured in three forest fragment areas of a large Amazonian city.

Subgenus/group (n)	Species	n (%)
*Nyssomyia* (5497)	*Lu. antunesi*⁎	5080 (65.46)
	*B. flaviscutellata*⁎	414 (5.33)
	*Lu. yulli yulli*	3 (0.04)
*Psychodopygus* (1707)	*Lu. davisi*⁎	1525 (19.65)
	*Lu. geniculata*	108 (1.40)
	*Lu. ayrozai*⁎	65 (0.84)
	*Lu. paraensis*⁎	5 (0.06)
	*Lu. hirsuta hirsuta*⁎	3 (0.04)
	*Lu. carrerai carrerai*	1 (0.01)
*Evandromyia* (248)	*Lu. infraspinosa*	242 (3.12)
	*Lu. brachyphala*	3 (0.04)
	*Lu. monstruosa*	2 (0.03)
	*Lu. pinotti*	1 (0.01)
Grupo *Oswaldoi* (56)	*Lu. rorotaensis*	52 (0.67)
	*Lu. trinidadensis*	4 (0.05)
Grupo *Aragoi* (112)	*Lu. barretoi barretoi*	111 (1.43)
	*Lu. aragoi*	1 (0.01)
*Vianniamyia* (41)	*Lu. tuberculata*	37 (0.48)
	*Lu. furcata*	4 (0.05)
*Trichopygomyia* (36)	*Lu. fongispina*	33 (0.42)
	*Lu. dasypodogeton*	3 (0.04)
*Sciopemyia* (25)	*Lu. sordelli*	25 (0.32)
*Lutzomyia* (18)	*Lu. gomezi*⁎	11 (0.14)
	*Lu. evangelista*	6 (0.08)
	*Lu. carvalhoi*	1 (0.01)
*Trichophoromyia* (15)	*Lu. ubiquitalis*⁎	15 (0.19)
*Psathyromyia* (2)	*Lu. lutziana*	2 (0.03)
*Micropygomyia* (2)	*Lu. micropyga*	2 (0.03)
*Pintomyia* (1)	*Lu. damascenoi*	1 (0.01)
Total (%)		**7760 (100.00)**

⁎ Species of medical importance for *Leishmania* sp.

**Fig. 1:**
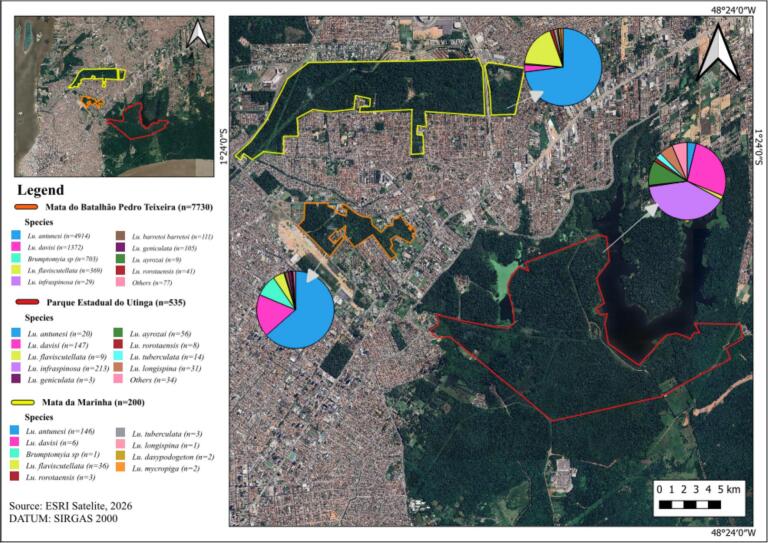
Spatial distribution and species composition of phlebotomine sandflies (Diptera: Psychodidae) captured across three urban forest fragments in the Belém metropolitan area, Pará, Brazil. Pie charts indicate the relative abundance of sand fly species recorded at each sampling site.

Species composition varied markedly among sampling areas, although a strong dominance pattern was observed across all sites (Table 3). *Nyssomyia antunesi* was the most abundant species, representing 60.01% of all captured specimens and occurring in the three forest fragments, with particularly high abundance in MBPT. The second most frequent species was *Ps. davisi* (18.01%), also recorded in all areas but showing higher abundance in MBPT and PEUT. *Brumptomyia* sp. França & Parrot, 1921 accounted for 8.33% of the captures and was almost exclusively recorded in MBPT. *Bichromomyia flaviscutellata* (4.89%) was present in all study areas, whereas *Evandromyia infraspinosa* (Mangabeira, 1941) (2.86%) was predominantly associated with PEUT. The remaining species occurred at low frequencies (<2%), many represented by only a few individuals. MBPT exhibited the highest species abundance and richness, concentrating the majority of dominant taxa, while PEUT contributed several intermediate-frequency species, and MM showed lower abundance but maintained the presence of the dominant vectors. Overall, the phlebotomine assemblage was characterised by strong dominance of a few species alongside a large number of rare taxa, a pattern typical of Amazonian forest environments. These compositional differences among sites are visually represented in Fig. 1, which displays georeferenced pie charts overlaid on a satellite image of the study area. The chart corresponding to MM is overwhelmingly dominated by *Ny. antunesi*, reflecting the low diversity and strong numerical dominance observed at that site. In contrast, the pie chart for MBPT shows a more complex composition, with visible contributions from *Ps. davisi*, *Brumptomyia* sp., *Psathyromyia barrettoi barrettoi* (Mangabeira 1942), *Ps. geniculatus* (Mangabeira, 1941), *Ps. ayrozai* (Barretto & Coutinho, 1940), and *Mi. rorotaensis* (Floch & Abonnenc, 1944), consistent with its higher species richness. The PEUT chart presents the most balanced distribution among the three sites, with proportional representation of several intermediate-frequency species alongside the dominant taxa, reflecting the greater diversity associated with this larger and better-preserved protected area (Table 4).

**Table 4:** Phlebotomine fauna captured in three forest fragments of the Belém metropolitan region, Pará state, Brazil, from 2014 to 2016. M: male; F: female.

Species	MBPT		PEUT		MM			
	F	M	F	M	F	M	Total	%
*Nyssomyia antunesi*	3660	1254	9	11	121	25	5080	60.01
*Psychodopygus davisi*	649	723	100	47	5	1	1525	18.01
*Brumptomyia* sp.	260	443	–	1	–	1	705	8.33
*Bichromomyia flaviscutellata*	213	156	7	2	19	17	414	4.89
*Evandromyia infraspinosa*	13	16	116	97	–	–	242	2.86
*Psathyromyia barrettoi barrettoi*	58	53	–	–	–	–	111	1.31
*Psychodopygus geniculatus*	34	71	1	2	–	–	108	1.27
*Psychodopygus ayrozai*	2	7	37	19	–	–	65	0.77
*Micropygomyia rorotaensis*	31	10	7	1	3	–	52	0.61
*Viannamyia tuberculata*	15	5	11	3	2	1	37	0.44
*Trichopygomyia longispina*	1	–	18	13	–	1	33	0.39
*Sciopemyia sordellii*	12	9	2	2	–	–	25	0.29
*Trichophoromyia ubiquitalis*	8	1	2	4	–	–	15	0.18
*Lutzomyia gomezi*	5	1	4	1	–	–	11	0.13
*Lutzomyia evangelistai*	2	–	4	–	–	–	6	0.07
*Psychodopygus paraensis*	2	3	–	–	–	–	5	0.06
*Micropygomyia trinidadensis*	4	–	–	–	–	–	4	0.05
*Viannamyia furcata*	1	–	3	–	–	–	4	0.05
*Evandromyia brachyphalla*	3	–	–	–	–	–	3	0.03
*Trichopygomyia dasypodogeton*	1	–	–	–	2	–	3	0.03
*Nyssomyia yuilli yuilli*	–	–	3	–	–	–	3	0.03
*Psychodopygus hirsutus hirsutus*	2	–	1	–	–	–	3	0.03
*Psathyromyia lutziana*	2	–	–	–	–	–	2	0.02
*Micropygomyia micropyga*	–	–	–	–	2	–	2	0.02
*Evandromyia monstruosa*	–	–	2	–	–	–	2	0.02
*Psathyromyia aragaoi*	–	–	1	–	–	–	1	0.01
*Lutzomyia carvalhoi*	–	–	1	–	–	–	1	0.01
*Psychodopygus carrerai carrerai*	–	–	–	1	–	–	1	0.01
*Pintomyia damascenoi*	–	–	1	–	–	–	1	0.01
*Evandromyia pinotti*	–	–	1	–	–	–	1	0.01
**Total (%)**	**4978**	**2752**	**331**	**204**	**154**	**46**	**8465**	**100,00**

A total of 205 pools comprising 2442 phlebotomine sand fly specimens collected from three urban forest fragments of the BMR were subjected to PCR analysis for *Leishmania* detection (Table 5). Most pools originated from MBPT (197 pools), while PEUT and MM contributed three and five pools, respectively. The majority of tested material corresponded to *Ny. antunesi*, followed by *Ps. davisi* and *Bi. flaviscutellata*. All analyzed pools tested negative for *Leishmania* DNA.

**Table 5:** Number of phlebotomine sand fly specimens and pools captured in the three urban forest fragments of the Belém metropolitan region, Pará state, Brazil, from 2014 to 2016, distributed by species.

Espécie	MBPT NE/NP	PEUT NE/NP	MM NE/NP	Total (NE/NP)
*Nyssomyia antunesi*	1754/73	–	36/2	1790/75
*Psychodopygus davisi*	422/84	–	–	422/84
*Bichromomyia flaviscutellata*	121/24	–	15/3	136/27
*Brumptomyia* sp.	58/5	–	–	58/5
*Sciopemyia sordelli*	4/2	–	–	4/2
*Evandromyia infraspinosa*	3/2	1/1	–	4/3
*Psychodopygus geniculatus*	6/1	–	–	6/1
*Viannamyia tuberculata*	4/1	2/1	–	6/2
*Psathyromyia barrettoi barrettoi*	4/1	–	–	4/1
*Micropygomyia rorotaensis*	4/1	–	–	4/1
*Micropygomyia trinidadensis*	4/1	–	–	4/1
*Lutzomyia gomezi*	1/1	–	–	1/1
*Evandromyia brachyphalla*	1/1	–	–	1/1
*Trichopygomyia longispina*	–	2/1	–	2/1
**Total**	**2386/197**	**5/3**	**51/5**	**2442/205**

NE: Number of specimens; NP: Number of pools; MBPT (Mata do Batalhão “Pedro Texeira”); PEUT (Parque Estadual do Utinga); MM (Mata da Marinha).

## 4 Discussion

This study demonstrates a high abundance and richness of phlebotomine sand flies in urban forest fragments located within the BMR. Although no *Leishmania* DNA was detected in the analyzed pools, the assemblage was strongly dominated by species recognised as confirmed or potential vectors, indicating that these environments maintain ecological conditions favourable for enzootic transmission cycles and potential human exposure.

Female predominance observed across all sampling sites is consistent with patterns commonly reported in CDC light-trap surveys conducted in Amazonian forests, reflecting behavioural differences related to host-seeking activity. The markedly higher abundance recorded in MBPT suggests that structurally heterogeneous forest environments subject to moderate anthropogenic disturbance may favour phlebotomine proliferation by increasing the availability of resting sites, organic substrates, and vertebrate hosts.

The community structure was characterised by strong dominance of a few taxa accompanied by numerous rare species, a pattern typical of Neotropical phlebotomine assemblages (Ready et al., 1997), *Nyssomyia antunesi* was overwhelmingly dominant, accounting for more than half of all captured specimens and occurring in all forest fragments. This species exhibits pronounced ecological plasticity and has been frequently associated with forest edges and anthropogenically modified environments, including urban forest remnants, reinforcing previous observations from Amazonian Brazil and neighbouring South American regions reporting similar dominance patterns in peri-urban forest ecosystems (Sánchez Uzcátegui et al., 2020; Pereira Júnior et al., 2019; Pinto et al., 2023; Carneiro et al., 2023; Barroso et al., 2025; Lamattina et al., 2024; Portella and Kraenkel, 2021; de Melo et al., 2024; Brasil. Ministério da Saúde. Secretaria de Vigilância em Saúde. Departamento de Vigilância das Doenças Transmissíveis, 2017; Brasil. Instituto Brasileiro de Geografia e Estatística, 2022; Galati, 2018; Ready et al., 1997; Fernandes et al., 1994; Paiva et al., 2007; Fonseca et al., 2021). These findings place the present results within a broader regional context in which urban forest fragments sustain vector assemblages comparable to those described in endemic areas across northern Brazil, Colombia, and other parts of tropical South America.

*Psychodopygus davisi*, the second most abundant species, also occurred in all sampling areas and has been widely reported in Amazonian transmission settings (Pereira Júnior et al., 2019; Ferreira et al., 2014; Gil et al., 2003; Souza et al., 2017). Likewise, *Bi. flaviscutellata*, although less abundant, was recorded in all sites and is recognised as a vector associated with rodent reservoirs and enzootic transmission cycles involving *L.* (*L.*) *amazonensis* (Carvalho et al., 2018; Ward et al., 1977; Rangel et al., 1996). The coexistence of these species suggests ecological continuity between preserved forest habitats and urban green areas.

Despite the high abundance of recognised or putative vector species, all analyzed pools tested negative for *Leishmania* DNA. This absence of parasite detection may be associated with seasonal variation in parasite circulation, since sand fly infection rates and host–vector contact intensity fluctuate according to rainfall patterns and humidity typical of equatorial climates, potentially resulting in temporal windows of low transmission during sampling. Additionally, the sampling period, although extensive, may not have coincided with peaks of enzootic amplification, which are often episodic in Amazonian environments. Natural infection rates in phlebotomines are typically very low, frequently below 1% even in endemic regions (Ready et al., 1997), meaning that large sample sizes or long-term monitoring are often required to detect infected individuals. Methodological factors may also have contributed, as pooled samples and low parasite loads can reduce PCR sensitivity and increase the probability of false-negative results, particularly when infections occur at low prevalence. The epidemiological scenario of BMR further supports this interpretation, as ATL occurs only occasionally and generally in focal patterns, indicating sporadic transmission rather than sustained urban outbreaks. Reported human cases in the municipality and surrounding areas have historically been associated with forest exposure or occupational activities near preserved environments, suggesting that transmission remains primarily sylvatic and spatially restricted (Gonçalves et al., 2020b).

Urban forest fragments function as biodiversity refuges embedded within a densely urbanised matrix, maintaining suitable microclimatic conditions such as high humidity, shaded environments, and abundant organic matter that favour sand fly development (Sánchez Uzcátegui et al., 2024). The selected sampling sites were chosen because they represent the principal preserved forest remnants within the metropolitan region, characterised by intense human circulation for recreation, military training, and research activities, thereby constituting interfaces where human–vector contact is most likely to occur. In addition, the limited but epidemiologically relevant dispersal capacity of phlebotomine sand flies allows individuals to move between forested habitats and adjacent anthropogenic environments, facilitating the spillover of sylvatic transmission cycles into urban settings (Neves et al., 2025). Longitudinal surveillance is fundamental for monitoring the emergence of local outbreaks, as well as their tendency towards large-scale epidemics in Amazonian populations. Considering that vector control health policies in Belém are not consistent or constant, favoring the emergence of phlebotomine sand flies without monitoring and tracking of leishmania throughout the seasons, this contributes to a scenario of widespread infection circulation. This is because in the Amazon, the world's largest tropical rainforest, the seasonal patterns favour the anticipation of outbreaks due to the emergence of vector populations. Monitoring measures for vector populations optimize the effectiveness of preventive interventions throughout the year, proving to be a cornerstone measure for the effective control of this disease, crucial for protecting public health and the efficient management of outbreaks. Such ecological interfaces are increasingly recognised as important settings for zoonotic disease emergence in tropical regions. Even in the absence of detected infections, high vector density may still represent epidemiological relevance, as transmission efficiency can be maintained when large vector populations coexist with infected reservoirs. Dipteran vectors readily adapt to anthropogenically modified environments, exploiting humid and shaded microhabitats present in the investigated areas (Carneiro et al., 2023; González et al., 2015).

This study presents some limitations that should be acknowledged. Only three urban forest fragments were included, which may not fully represent the environmental heterogeneity of the metropolitan region. In addition, the cross-sectional design limited the temporal assessment of transmission dynamics, and the sensitivity of PCR-based detection, particularly when applied to pooled samples, may have reduced the likelihood of detecting infected specimens. No inferential statistical tests were performed to compare sites or species, as the study was designed as a descriptive survey focused on faunal composition and relative abundance patterns; consequently, interpretations were based on proportional distributions and observed ecological trends rather than hypothesis-testing approaches. Future longitudinal and spatially expanded investigations would contribute to a better understanding of seasonal infection dynamics and parasite circulation.

## 5 Conclusion

Overall, the results demonstrate that urban forest remnants in the BMR sustain diverse and abundant phlebotomine assemblages dominated by species of recognised medical importance. Although no *Leishmania* DNA was detected in the analyzed samples, these environments remain ecologically suitable for the maintenance of transmission cycles and therefore represent potential risk areas requiring continuous entomological surveillance and ecological monitoring. The urban forest fragments investigated harbour a broad phlebotomine fauna, with marked dominance of *Ny. antunesi*, *Ps. davisi*, and *Bi. flaviscutellata*, species recognised as confirmed or suspected vectors of *Leishmania*. Their occurrence in forested environments characterised by frequent human activity and the presence of wild vertebrate hosts highlights ecological interfaces favourable to American tegumentary leishmaniasis transmission. These findings reinforce the importance of sustained surveillance and integrated monitoring of sand fly populations to support prevention strategies and improve understanding of transmission dynamics in urbanised Amazonian landscapes.

## CRediT authorship contribution statement

**Yago Costa Vasconcelos dos Santos:** Visualization, Methodology, Investigation, Formal analysis, Data curation. **Thiago de Matos Bezerra:** Formal analysis. **Doralice Crisóstomo dos Reis:** Formal analysis. **Rogério Valois Laurentino:** Writing – original draft. **Leonardo Miranda dos Santos:** Writing – review & editing, Writing – original draft, Conceptualization. **Ana de Nazaré Martins da Silva:** Methodology. **Thiago Vasconcelos dos Santos:** Validation, Investigation. **Edna Aoba Yassui Ishikawa:** Writing – original draft, Validation, Supervision, Methodology, Investigation, Data curation, Conceptualization.

## Funding

The publication of this article was funded through an agreement with the 10.13039/501100002322Coordenação de Aperfeiçoamento de Pessoal de Nível Superior (CAPES), Brazil.

## Declaration of competing interest

The authors declare no conflicts of interest.
